# Phenethyl Isothiocyanate-Containing Carbomer Gel for Use against Squamous Cell Carcinoma

**DOI:** 10.3390/pharmaceutics13010106

**Published:** 2021-01-15

**Authors:** Ositomiwa O. Osipitan, Yi Shi, Anthony J. Di Pasqua

**Affiliations:** 1 Department of Pharmaceutical Sciences, School of Pharmacy and Pharmaceutical Sciences, Binghamton University, 96 Corliss Ave., Johnson City, NY 13790, USA; oosipit1@binghamton.edu (O.O.O.); yishi.pharmsci@gmail.com (Y.S.); 2 Department of Biomedical Engineering, The Thomas J. Watson College of Engineering and Applied Science, Binghamton University, 4400 Vestal Pkwy. E., Binghamton, NY 13902, USA

**Keywords:** phenethyl isothiocyanate, gel dosage form, topical application, skin permeability, skin cancer

## Abstract

It is currently estimated that one in every five Americans will develop skin cancer during their lifetime. Squamous cell carcinoma (SCC) is a common type of skin cancer that can develop due to the skin’s exposure to the sun. Herein, we prepared a topical gel containing 0.5% *v*/*w* phenethyl isothiocyanate (PEITC) for the treatment of SCC. PEITC is a naturally occurring isothiocyanate that has been shown to have efficacy against various types of cancer in preclinical studies. We first incorporated PEITC into a carbomer gel. A uniform formulation was prepared, and its viscosity was appropriate for topical application. We then demonstrated the release of PEITC from the gel into and through a Strat-M skin-like membrane. Finally, the effects of the PEITC-containing gel were tested against SCC and normal keratinocytes skin cells in culture, and these results were compared to those obtained for free 5-fluoruracil (5-FU), a commonly used skin-cancer drug. Our results show that a homogeneous PEITC-containing topical gel can be prepared and used to kill SCC cells. Thus, our formulation may be useful for treating SCC in the clinic.

## 1. Introduction

Isothiocyanates (ITCs) are organic compounds with a –N=C=S functional group that can react with cellular nucleophiles. Naturally occurring ITCs are contained in cruciferous vegetables (e.g., broccoli, watercress, etc.) as glucosinolates and are released via hydrolysis by the enzyme myrosinase. Myrosinase is also contained in these vegetables and in the human gut. ITCs have been widely investigated as potential cancer chemopreventive and therapeutic agents; they may prevent tumor initiation, promotion, and progression [1]. Studies have suggested that the covalent binding of ITCs to certain protein targets may play an important role in ITC-induced apoptosis and cell growth inhibition [2]. For example, the ability of ITCs to bind to cysteine residues in tubulin leads to conformational changes that result in apoptosis of cancer cells [3]. Phenethyl isothiocyanate (PEITC) and benzyl isothiocyanates have been found to inhibit the activation of carcinogens by inhibiting certain phase I enzymes [4]. In an in vivo study, CYP cytochrome P450s enzymes were inhibited, and cancer development was decreased [1,4,5]. PEITC and sulforaphane, another type of ITC, were found to decrease the DNA binding of nuclear factor kappa B (NF-κB), which is an ubiquitously expressed proinflammatory factor that plays an important role in tumor-cell development and growth, angiogenesis, and survival [4,6]. Investigators have previously concluded that dietary intake of ITCs is associated with reduced risk of colorectal cancer in humans [7], and other studies have shown associations with a high intake of cruciferous vegetables and a lower risk of cancers, including both lung and colorectal [4].

PEITC is a naturally occurring ITC, and gluconasturtiin, the precursor of PEITC, is found in watercress. PEITC has been shown to have efficacy against various cancers in preclinical animal models [8]. Micromolar concentrations of PEITC have been shown to be therapeutic in the laboratory, and micromolar concentrations of ITCs can be safely maintained in the blood of humans after oral administration [9]. PEITCs have been formulated in liposomal nanoparticle for delivery to nonsmall cell lung cancer cells [10,11,12]. As a result of its fairly lipophilic nature and poor aqueous solubility, liposomes are an appropriate delivery method. Seema and coworkers also proposed graphene oxide as a potential nanocarrier for PEITC [13]. Thus, there is a strong rationale for studying the use of PEITC against a wide variety of cancers [14], including skin cancer.

It is estimated that approximately one in five Americans will develop skin cancer at some point in their lives by the age of seven [15]. The two major forms of nonmelanoma skin cancer (NMSC) are basal cell carcinoma (BCC), in the deepest layer of the epidermis, and squamous cell carcinoma (SCC), in cells just beneath the outermost layer of the epidermis. In the US, approximately 1.3 million NMSC cases are diagnosed annually [16]. Over the past few decades, the cases of SCC and BCC have continually increased in the US by 3–10% and 20–80% per year, respectively [17].

Recently, Lam-ubol et al. demonstrated that PEITC is toxic toward the human SCC-25 cell line [18]. After 72-h exposure, PEITC was more toxic toward these tongue-derived SCC cancer cells than oral nontumorigenic keratinocytes, with IC_50_′s of 8 µM and 21.5 µM, respectively. Although this SCC cancer cell line was derived from tongue epithelial cells, they have also been used to develop a 3D in vitro skin cancer model [19]. Thus, we herein prepared a homogeneous PEITC-containing carbomer gel; tested its uniformity, viscosity and ability to release PEITC; and studied its toxicity against human SCC-25 cells and keratinocytes. We then compared its efficacy to free 5-fluorouracil (5-FU), as 5-FU is sometimes used in the clinic against certain skin cancers [20,21].

## 2. Materials and Methods

### 2.1. Materials

PEITC was purchased from Sigma Aldrich (St. Louis, MO, USA); 5-Fluorouracil was purchased from TCI America (Portland, OR, USA); and 1,2-benzenedithiol (BDT) was purchased from VWR (Radnor, PA, USA). Carbopol 940 and triethanolamine were purchased from Fisher Scientific (Hampton, NH, USA), and dimethyl sulfoxide (DMSO) was from VWR (Radnor, PA, USA). The MTS assay kit was purchased from Promega (Madison, WI, USA), and human SCC-25 and keratinocytes were purchased from the American Type Culture Collection (ATCC) (Manassas, VA, USA).

### 2.2. Cell Culture

The human SCC-25 cells were cultured in a DMEM/Ham’s F12 medium purchased from Sigma Aldrich (St. Louis, MO, USA) and were supplemented with 10% fetal bovine serum (FBS), 0.4 µg/mL hydrocortisone, 15 mM HEPES, sodium bicarbonate, and L-glutamine, all of which were also purchased from Sigma Aldrich. The culture medium was changed every 2–3 days, and the cells were subcultured at 70–80% confluency. The human primary epidermal keratinocytes were cultured in a dermal cell basal medium from the ATCC and were supplemented with penicillin-streptomycin-amphotericin B, phenol red, and a supplemental kit, as recommended by the ATCC. The culture medium was again changed every 2–3 days and subcultured at 70–80% confluency.

### 2.3. Formulation of the Carbomer Gel Containing 0.5% PEITC

Carbomer concentrations (0.5–2%) were tested to determine the best base. The 0.5% stock was chosen due to its viscosity. Carbopol 940 (0.05 g) was dispersed in 10 mL of deionized water. The mixture was kept at room temperature for 24 h to form a 0.5% *w*/*v* carbomer gel. PEITC (0.05 mL) was dissolved in 0.025 mL of DMSO, as PEITC is more soluble in DMSO than in water, and DMSO is miscible with the carbomer gel. This solution was then added to 5 mL of the 0.5% *w*/*v* carbomer gel, reducing the total carbomer concentration to 0.49% *w*/*v*. Finally, triethanolamine (10 µL) was added to the gel mixture, and the gel was mixed via probe sonication at an amplitude of 20 MHz for 10 s and was then vortexed for 1 min and gently stirred. A blank carbomer gel was prepared in the same manner but without adding PEITC. The pH of both gels was measured using a Mettler Toledo F20 pH-meter. 

### 2.4. Homogeneity Tests

The concentration of PEITC in each PEITC carbomer gel was determined using the 1,2-benzenedithiol-based cyclocondensation assay [22]. First, an 8 mM BDT in methanol solution was prepared. Four samples (60–65 mg each) were collected from different areas of the gel, and the samples were dissolved in the recommended buffer of potassium phosphate, pH 8.5. An aliquot of the sample (40 μL), 960 μL of phosphate buffer (pH 8.5), and 1000 μL of BDT/methanol solution were mixed and heated for 2 h in glass vials. The solution was allowed to cool at room temperature prior to an absorbance reading at λ = 365 nm using a Spectra i3x plate reader, and a PEITC standard curve (*R*^2^ = 0.98) was used to determine the concentrations. For the standard curve, PEITC was first solubilized in DMSO and then mixed with potassium phosphate buffer. This stock was further diluted to 1:100 in potassium phosphate buffer. The final concentrations were then made in the phosphate buffer, and solubilized BDT in methanol was added to determine the PEITC concentrations. PETIC should be stored at 2–8 °C [23]; thus, we kept the formulation at these temperatures during storage. The stability of the formulation under these storage conditions was determined after 30 and 55 days.

### 2.5. Viscosity Tests

The viscosities of the formulations were determined using a microVISC from RheoSense (San Ramon, CA, USA) at room temperature (20–25 °C) and at skin temperature (32 and 37 °C). After formulating a PEITC carbomer gel, the viscosity measurements were performed. The same study was performed using the blank carbomer gel and a commercially available gel, Alocane. Alocane (Quest Products, Inc., Pleasant Prairie, WI, USA) contains 4% Lidocaine HCl and the following inactive ingredients: 1,3-propanediol, aloe barbadensis leaf juice, dimethyl isosorbide, hydroxyethyl cellulose, phenoxyethanol, chlorphenesin, capryly glycol, tocopheryl acetate, and water, and has a pH of 5.3. Using the provided pipette for the instrument, 10 μL of sample were injected into the viscometer for each viscosity measurement, and the viscosity was measured at shear rates of 10 to 70 s^−1^ at 25, 32 and 37 °C. Samples were run in triplicate.

### 2.6. Transdermal Diffusion Tests

Transdermal diffusion tests were conducted using a Franz cell with a 11.28 mm orifice diameter and Millipore Strat-M membranes, a type of membrane commonly used in predictive studies for diffusion in human skin [24,25]. Franz cell systems are appropriate for evaluating uptake into and through such membranes [26]. For each data point, a portion of the gel formulation (0.1 g) was applied onto the membrane in the donor chamber, and the receptor chamber was filled to its capacity (8 mL) with phosphate buffer (pH 8.5). The system was sustained at 37 °C using a VWR Heated Re-circulator, Model 1104 (Radnor, PA, USA), and the solution was agitated using a magnetic stir bar at 400 rpm. The amount of PEITC in both the gel and reservoir was determined using a cyclocondensation assay and a UV–visible spectroscopy, as described earlier. We studied the PEITC content in the gel and receptor media at 15, 30, 60, 120, and 300 min, and this was performed in triplicate. The standard curve that we used was prepared as described above. PEITC was first solubilized in DMSO and then mixed with potassium phosphate buffer. This stock was further diluted to 1:100 in potassium phosphate buffer. The final concentrations were then made in the phosphate buffer, and solubilized BDT in methanol was added to determine the PEITC concentrations (*R*^2^ = 0.98).

### 2.7. PEITC Carbomer Gel and 5-FU Cell Viability Tests

Human SCC-25 cells and keratinocytes were seeded in 12-well plates at approximately 5 × 10^4^ cells/mL in 1.5 mL complete culture medium, per well. After 24 h, the cells, at approximately 50% confluency, determined visually at 10× using an Olympus inverted microscope, were treated with PEITC carbomer gel or blank carbomer gel at the corresponding weight (*n* = 3 for each dose; ≤15 mg gel used per dose).

The gels were placed on inserts above the cells, and PEITC was released through the insert and into the culture medium. The inserts used in the toxicity studies were made of a thin polycarbonate membrane with 0.4 μm pores. The PEITC concentrations in culture medium after 30 min were determined beforehand using inserts and the previously described cyclocondensation reaction. After 30 min, the inserts were removed, and the culture medium changed. As FBS was not present in the keratinocyte culture medium, treatment of both SCC and keratinocytes was carried out in the absence of FBS. Thus, during the 30-min treatment, FBS was not included in the SCC medium, but it was present at all other times. To determine the effects of FBS on the toxicity of PEITC toward SCC cells, a separate study was performed with FBS present during the 30-min treatment time. In all cases, after a 24-h recovery in a complete culture medium, an MTS assay was performed to determine cell viabilities. Cells were incubated with MTS for 2 h, then 100 µL of each sample was taken in triplicate, placed in 96-well plates, and a Spectra i3x plate reader used to read the absorbance at λ = 490 nm. 5-FU studies were performed similar to above; however, a 30-min exposure with a 24-h recovery showed no toxicity. Thus, a 48-h continuous exposure study with 5-FU was performed in both cell lines. This was to test the free drug, so no inserts were used. 5-FU was solubilized in PBS and used as such. For all cell studies, besides the 30-min 5-FU cytotoxicity study, the dose-response curves were generated a second time to confirm trends, again with *n* = 3 for each dose (in Supplementary Materials). 

### 2.8. Analysis of Diffusion and Cell Viability Data

Excel’s Solver program was used to generate curves for the diffusion and cell studies. All the concentrations leading to 50% cell proliferation inhibition (IC_50_) were calculated using the following equation in Solver:

(1) $$\mathrm{Percent}\;\mathrm{cell}\;\mathrm{viability}=\left(100-b\right)*e^{-a*\mathrm{concentration}}+b$$

where *a* and *b* were parameters determined by the program [27]. When using Solver, a best fit curve is created using the square of the difference between the experimental data and predicted data. All experimental points are presented with said best fit curves in the text or supporting information.

## 3. Results

### 3.1. Preparation and Characterization of the Gel

**Figure 1:**
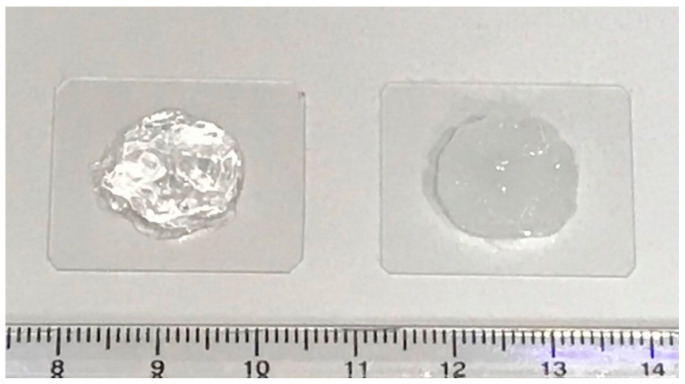
Photographic image of the blank carbomer (left) and phenethyl isothiocyanate (PEITC) carbomer (right) gels.

As is shown in Figure 1, the blank carbomer gel is clear, whereas the PEITC carbomer gel is a uniform, off-white semisolid. This is to be expected as PEITC has a light yellow hue. Table 1 shows the various components used to produce the gel. The pH values for the blank carbomer and the PEITC carbomer gels were 6.0 and 6.3, respectively. Such pH values are appropriate for the topical application to the skin [28]. The gels prepared consistently contained 0.5% *v*/*w* PEITC (Table 2). To determine if PEITC was evenly distributed throughout each gel, four aliquots from various areas of the semisolid were collected, and the PEITC concentrations were determined using the cyclocondensation assay. The uniformity results for each of the PEITC gels used in this study are shown in Table 2. Each value is the average (with standard deviation) of values obtained for each gel. From these data, it can be concluded that all are relatively uniform within themselves, with deviations of 8.2% or less. To test stability, a PEITC carbomer gel was stored at 4 °C, and then the homogeneity test was performed again at *t* = 30 and 55 days. At day 30, we measured 29.8 ± 4.1 mg, and at day 55, we measured 26.6 ± 1.9 mg. These data are similar to those collected at *t* = 0, meaning the formulation is relatively stable at 4 °C, which is the temperature that PEITC should be stored [23]. Furthermore, no color change was observed, and the gels handled similarly to before.

**Figure 2:**
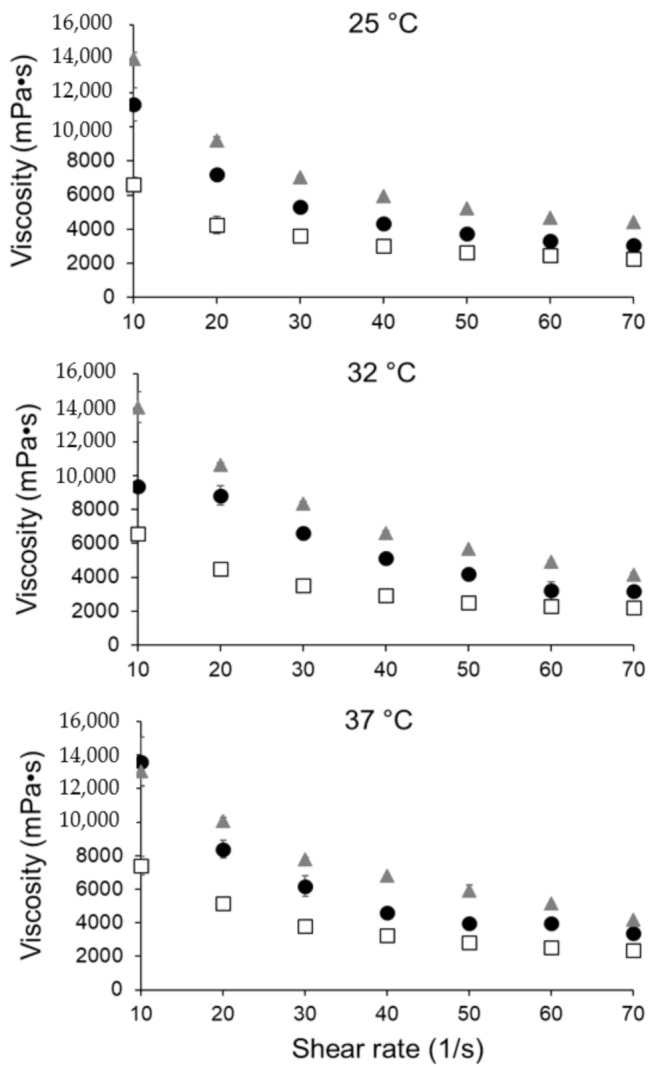
Viscosity of PEITC carbomer gel (gray triangles), blank carbomer gel (white squares), and commercially available Alocane gel (black circles) at 25, 32, and 37 °C. All samples run in triplicate.

Viscosities of the blank carbomer gel, PEITC carbomer gel, and commercial Alocane gel were measured at temperatures of 25, 32, and 37 °C at various shear rates. The skin surface has been reported to range from 32 to 37 °C [29,30]; thus, viscosities at 32 and 37 °C are relevant. These values are shown in Figure 2. The PEITC carbomer gel had viscosities similar to the commercially available Alocane gel and was comparable to the viscosity of a commercially available 5-FU cream [31]. Altogether, these data demonstrate that the PEITC carbomer gel would be appropriate for spreading and staying on the skin.

**Figure 3:**
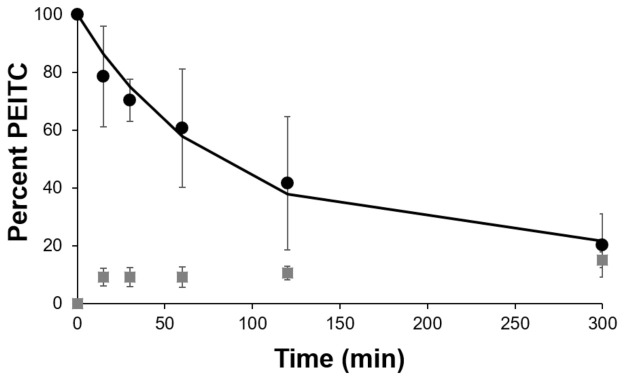
Percent phenethyl isothiocyanate (PEITC) in gel (black circle markers and the black line) and in receptor (gray square markers) over time, when on a STRAT-M membrane in a Franz cell at 37 °C.

The ability of the PEITC in the gel to permeate into and through the skin was then determined using a Strat-M synthetic membrane, which serves as a model for human skin [24]. We performed these studies at 37 °C, based on studies reported in the literature [32,33,34]. The membrane was placed onto the diffusion system and we studied the release of PEITC over a 5-h period. Figure 3 shows the percentage of PEITC retained in the gel at various time points and that found in the receptor chamber. These values were determined using the cyclocondensation assay and UV-visible spectroscopy. We observed a sustained release of PEITC over this period, and approximately 80% was released from the gel after 5 h. We presume that the remainder of PEITC at each time point (that not in the gel or receptor) was retained in the membrane, as this was a closed system.

### 3.2. Efficacy of PEITC Carbomer Gel in Cell Culture

**Figure 4:**
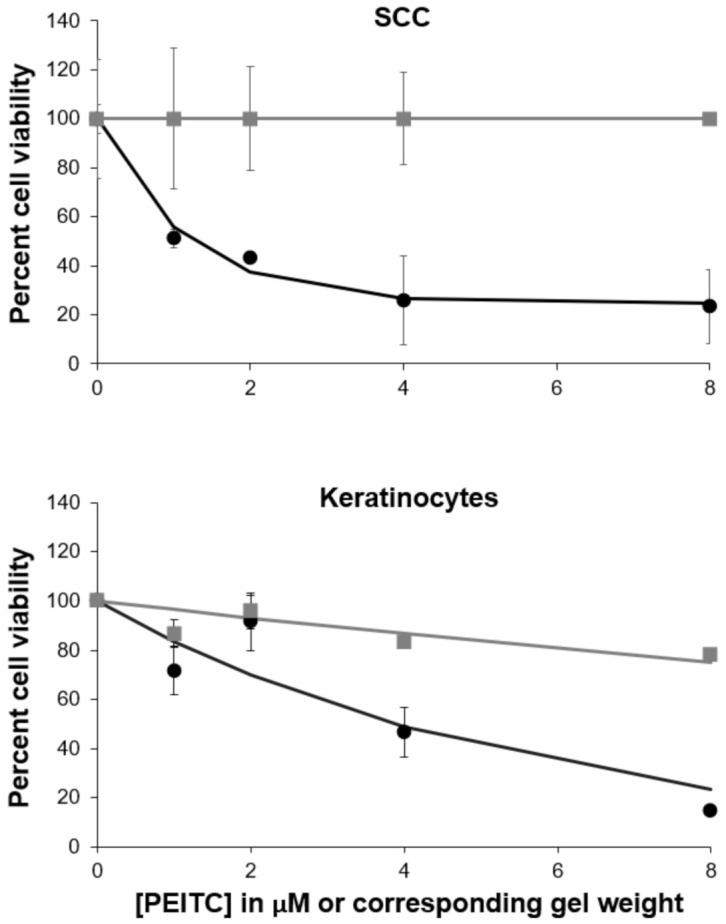
Dose-responses with the treatment of phenethyl isothiocyanate (PEITC) carbomer gel at concentrations of 1, 2, 4, and 8 µM PEITC and blank carbomer gel at corresponding masses. Blank carbomer gel toxicities are shown with gray square markers and the gray line, and PEITC carbomer gel toxicities are shown with black circle markers and the black line.

**Figure 5:**
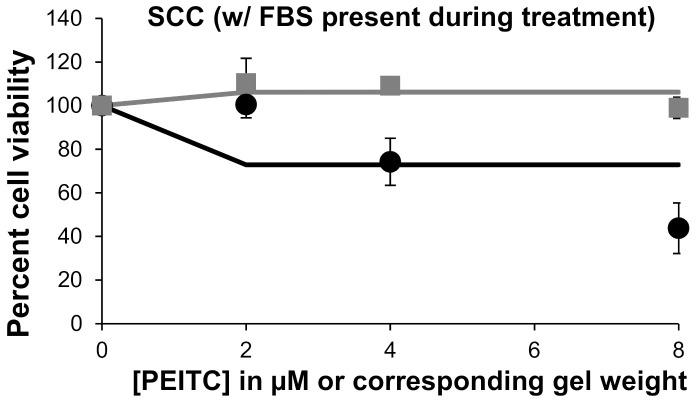
Dose-responses with treatment of phenethyl isothiocyanate (PEITC) carbomer gel at concentrations of 2, 4, and 8 μM PEITC and blank carbomer gel at corresponding masses. Blank carbomer gel toxicities are shown with gray square markers and the gray line, and PEITC carbomer gel toxicities are shown with black circles and the black line. These SCC-25 cells were treated with the presence of FBS.

The efficacy of the PEITC-containing carbomer gel was tested in a cell culture. Using 12-well plates, the gel was placed on inserts, and PEITC passed through the insert membranes to the cells below. We first determined that 31.5 µg of PEITC was contained in the reservoir after a 30-min exposure to 0.1 g gel. On the basis of this value, the amount of gel required for concentrations of 1–8 µM PEITC were calculated. The 30-min time point was chosen because it represented a reasonable therapy window for a patient, and the amount of gel used was reasonable for real-life applications. The weight equivalents of the blank carbomer gel were also studied in a cell culture. The blank carbomer gel was only slightly toxic toward both cell lines, although it appears that keratinocytes were more sensitive to it than the SCC-25 cells (Figure 4). As was expected, exposure to PEITC was toxic to human SCC, and a dose-response was observed. A similar trend was observed when keratinocytes were exposed to PEITC. From the data shown in Figure 4, for cells treated with 1, 2, 4, and 8 μM PEITC, IC_50_ values of 1.2 μM and 3.8 μM were calculated for SCC-25 cells and keratinocytes, respectively. When FBS was present in a culture medium during the 30-min treatment of SCC cells with PEITC, reduced toxicity was observed (Figure 5). The Solver-generated best fit curve plateaus at approximately 75% cell viability, demonstrating that FBS inactivates some PEITC, making it less applicable for therapy. This is consistent with the literature, which states that PEITC binds to proteins [2]. 

**Figure 6:**
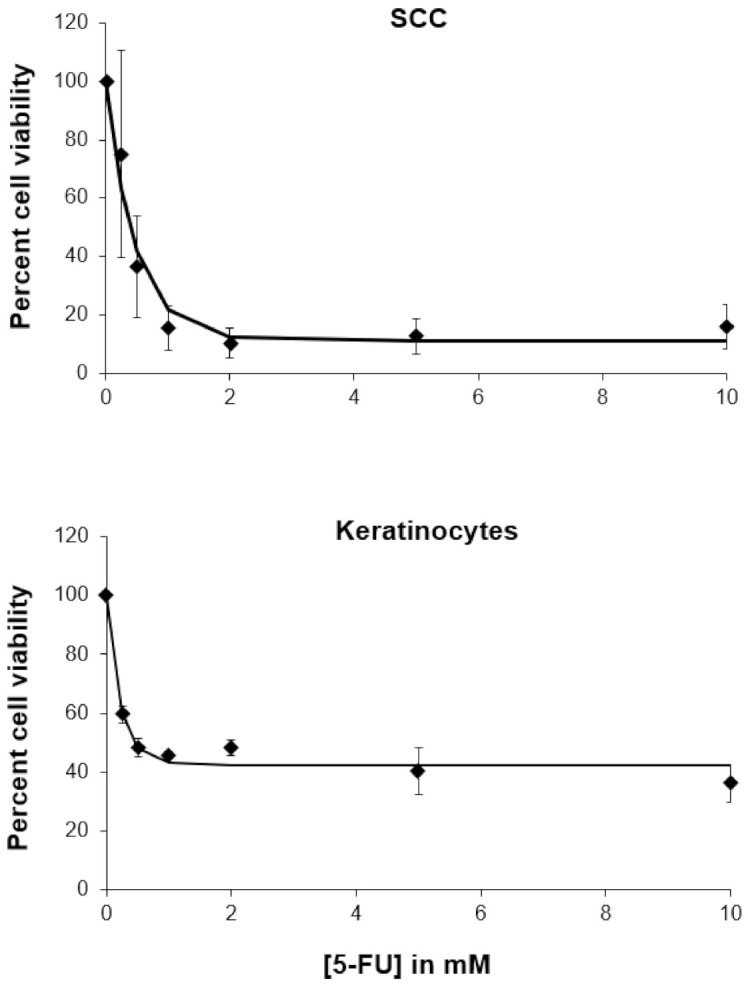
Dose-responses with the treatment of 5-FU for a 48-h continuous exposure.

We then tested the toxicity of 5-FU toward these cell lines. Levy et al. determined that a total of 2.2% of 5-FU in a 5% 5-FU formulation is absorbed [35]. From this, we estimated what the concentration of 5-FU released from 100 mg of gel would be. This concentration was approximately 0.5 mM. We decided to test the doses below and above this estimate. When the cell studies were performed under the same conditions as the aforementioned PEITC carbomer gel studies (i.e., 30-min exposure time and a 24-h recovery), 5-FU at concentrations of up to 5 mM did not significantly decrease viability in either cell line (in Supplementary Materials). It was previously shown that 24-h exposure to 5-FU had no effect on either SCC-25 or human epidermal cells [36]. Thus, a 48-h continuous exposure study was performed in the absence of FBS (Figure 6). The IC_50_ values of 5-FU against SCC-25 cells and keratinocytes were both approximately 0.4 mM after a 48-h exposure. A decrease in viability over 48 h is most likely due to 5-FU interfering with the S phase of the cell cycle [36].

## 4. Discussion

We herein incorporated PEITC into a topical carbomer gel for use against SCC lesions. We chose to prepare a gel with a low percentage of PEITC, as only low concentrations of PEITC are needed to elicit an effect in vitro and in vivo [9]. We prepared a formulation that releases adequate amounts of PEITC in a short period of time. This same formulation was prepared four times. The PEITC content and the uniformity of PEITC in the gel were similar in all four attempts. Furthermore, its pH of 6.3 and its viscosity at temperatures 25–37 °C is optimal for the application to skin [28,31]. The ability of PEITC to penetrate into and through the Strat-M membrane demonstrates its potential for treating SCC in the clinic. SCC is located near the surface of the skin [37], thus PEITC retained in this region for long periods of time increases the exposure of SCC to therapeutic concentrations of PEITC. The diameter of the Strat-M membrane used for these permeability studies is comparable to that of a Stage II SCC lesion [38,39].

Recently, Lam-ubol et al. demonstrated that PEITC is toxic toward SCC-25 cells [18]. The toxic effects of PEITC could be due to a number of factors, including oxidative stress [18] and binding to cellular proteins [3,40,41]. This SCC-25 cell line was also used by Brauchle et al. to develop a 3D skin-tissue model for studying disease progression and therapy [19]. Thus, we tested our formulation against this SCC cell line and human keratinocytes. Unlike 5-FU, which needed to be present in culture medium for 48 h to have effects, μM concentrations of PEITC released over 30 min were sufficient to kill SCC cells. Collectively, these data suggest that only a small amount of PEITC carbomer gel would need to be applied to a SCC lesion for 30 min to sufficiently kill SCC cells. However, since the formulation is also toxic to keratinocytes, exposure to healthy skin should be minimized.

The drug 5-FU is currently used against SCC of the skin and is administered orally or, more commonly, via a topical cream [20]. It is known for having toxic side-effects [42]; these side-effects include allergic contact dermatitis, erosions, hyperpigmentation, hypopigmentation, irritant dermatitis, onycholysis, and others [43]. 5-FU’s toxic side-effects are more severe in patients with dihydropyrimidine dehydrogenase (DPD) deficiency, which 3–5% of the general population has [44]. Work continues to be done on preparing an optimal topical formulation of 5-FU. In 2015, Khan and coworkers prepared a novel 5-FU-containing carbomer gel that had improved skin penetration and skin deposition of drug, compared to the marketed 5-FU formulation [45]. However, there is still room for alternative SCC treatments, such as the one described here. Furthermore, besides the ability of PEITC to kill cancer cells directly, it also has the ability to sensitize cancer cells to radiation [46]. Thus, this gel may be useful in the clinic for use with current brachytherapy approaches and future radiotherapeutic modalities [47,48]. Future work in this area should be explored.

## 5. Conclusions

In this work, our goal was to formulate a gel that could be used to treat SCC using naturally occurring isothiocyanates. We previously used PEITC to treat lung cancer cells and showed a reasonable therapeutic window [11]. The blank carbomer gel base used here was only slightly cytotoxic to SCC and keratinocytes. Naturally occurring PEITC at small concentrations was able to elicit a therapeutic effect. These preliminary findings suggest that in vivo study is warranted.

## Figures and Tables

**Table 1:** Phenethyl isothiocyanate (PEITC) carbomer gel formulation parameters.

PEITC (mL)	Carbopol 940 (g)	DMSO (mL)	Triethanolamine (mL)	Water (mL)
0.05	0.025	0.025	0.01	5.00

**Table 2:** Homogeneity results for phenethyl isothiocyanate (PEITC) carbomer gels ^1^.

Gel	PEITC per Gel (mg ± STD)
1	26.0 ± 1.4
2	30.7 ± 2.4
3	25.6 ± 2.1
4	23.6 ± 1.8

^1^ Each gel weighed 5.1 g.

## Data Availability

Data supporting the results can be obtained by contacting the authors.

## Supplementary Materials

The following are available online at https://www.mdpi.com/1999-4923/13/1/106/s1, Figure S1: Dose-responses with treatment of phenethyl isothiocyanate (PEITC) carbomer gel at concentrations of 2, 4, and 8 µM PEITC and blank carbomer gel at corresponding masses, Figure S2: Dose-responses with treatment of phenethyl isothiocyanate (PEITC) carbomer gel at concentrations of 2, 4, and 8 µM PEITC and blank carbomer gel at corresponding masses, Figure S3: Dose-responses with treatment of 5-FU for 30-min continuous exposure and 24-h recovery, Figure S4: Dose-responses with treatment of 5-FU for 48-h continuous exposure.
