# Supplementary material for: Phenethyl Isothiocyanate-Containing Carbomer Gel for Use against Squamous Cell Carcinoma
Source: Pharmaceutics. 2021 Jan 15;13(1):106. doi: 10.3390/pharmaceutics13010106 (PMC7830123; doi:10.3390/pharmaceutics13010106)
Supplement: Supplementary file 1 [file pharmaceutics-13-00106-s001.pdf]

pharmaceutics

Supplementary Materials: Phenethyl Isothiocyanate-Containing Carbomer Gel for Use Against Squamous Cell Carcinoma

Ositomiwa O. Osipitan, Yi Shi and Anthony J. Di Pasqua

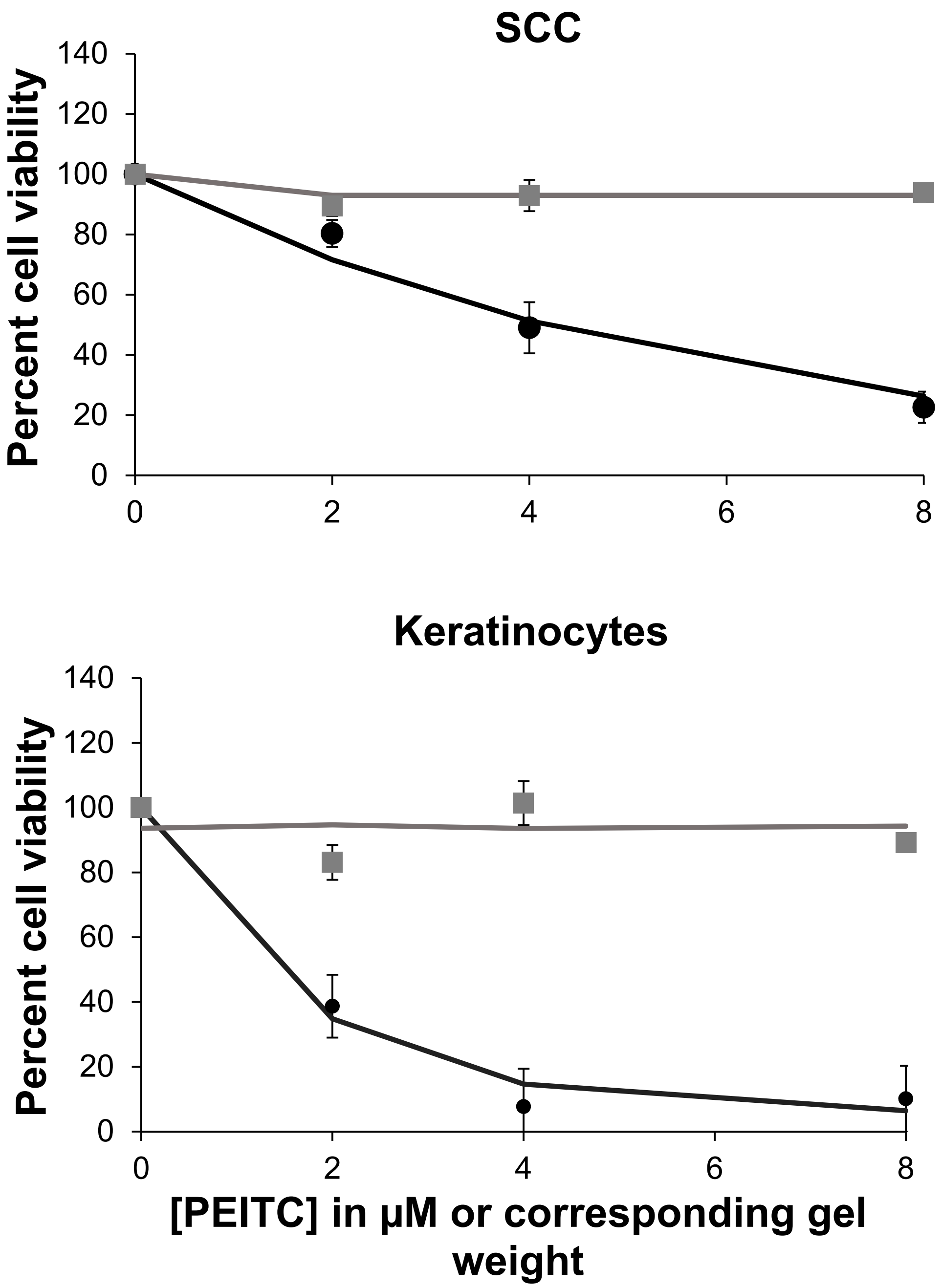

Figure S1. Dose-responses with treatment of phenethyl isothiocyanate (PEITC) carbomer gel at concentrations of 2, 4, and 8 μM PEITC and blank carbomer gel at corresponding masses. Blank carbomer gel toxicities shown with gray square markers and a gray line, PEITC-carbomer gel toxicities shown with black circles and a black line.

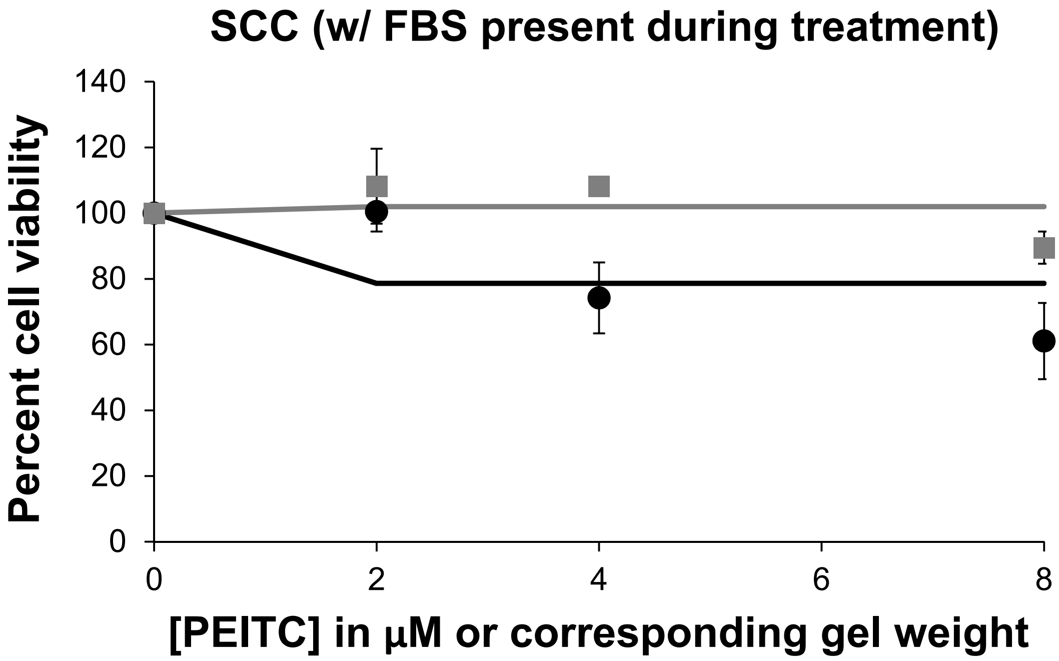

Figure S2. Dose-responses with treatment of phenethyl isothiocyanate (PEITC) carbomer gel at concentrations of 2, 4, and 8 µM PEITC and blank carbomer gel at corresponding masses. Blank carbomer gel toxicities shown with gray square markers and a gray line, PEITC-carbomer gel toxicities shown with black circles and a black line.

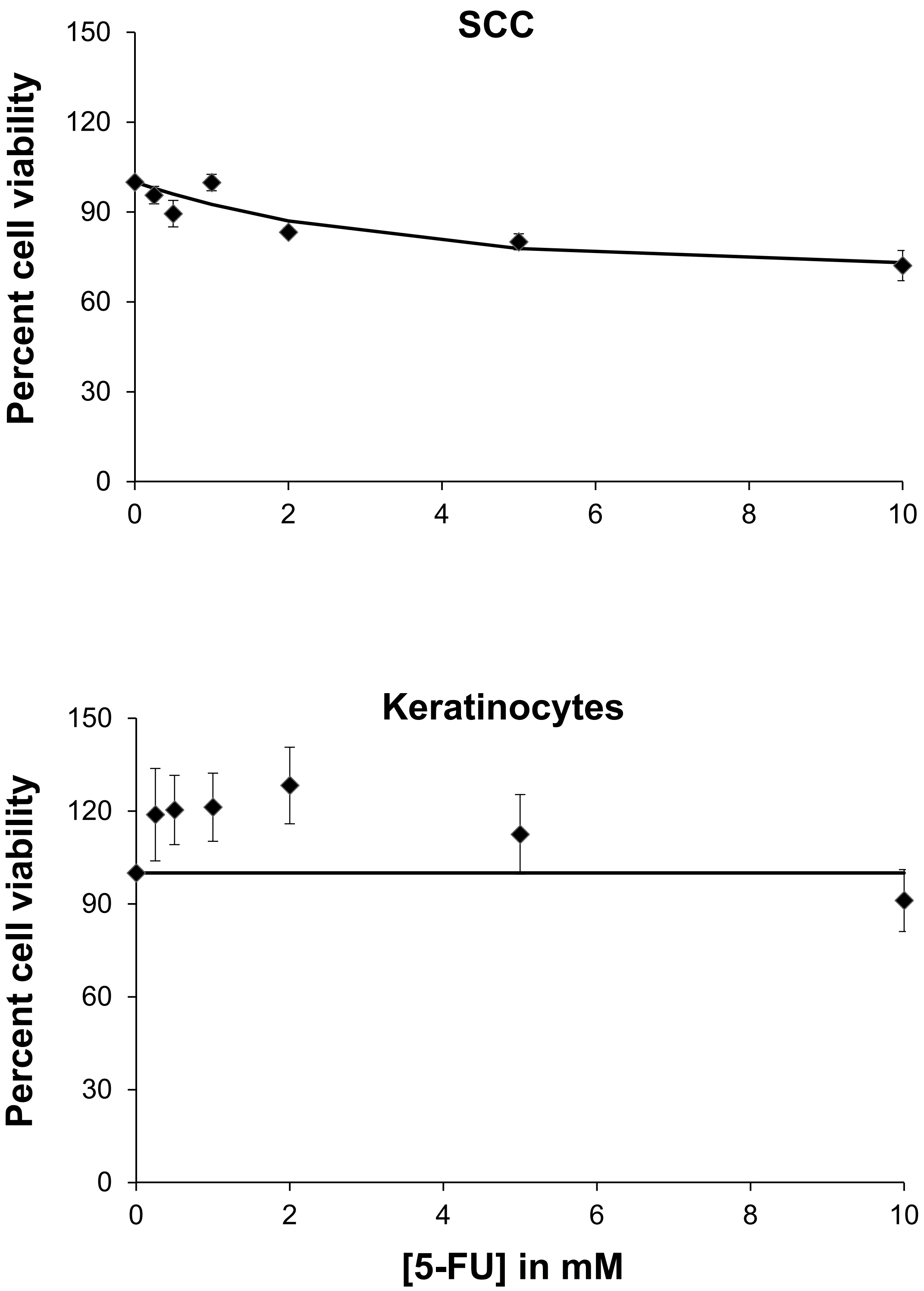

Figure S3. Dose-responses with treatment of 5-FU for 30-min continuous exposure and 24-h recovery.

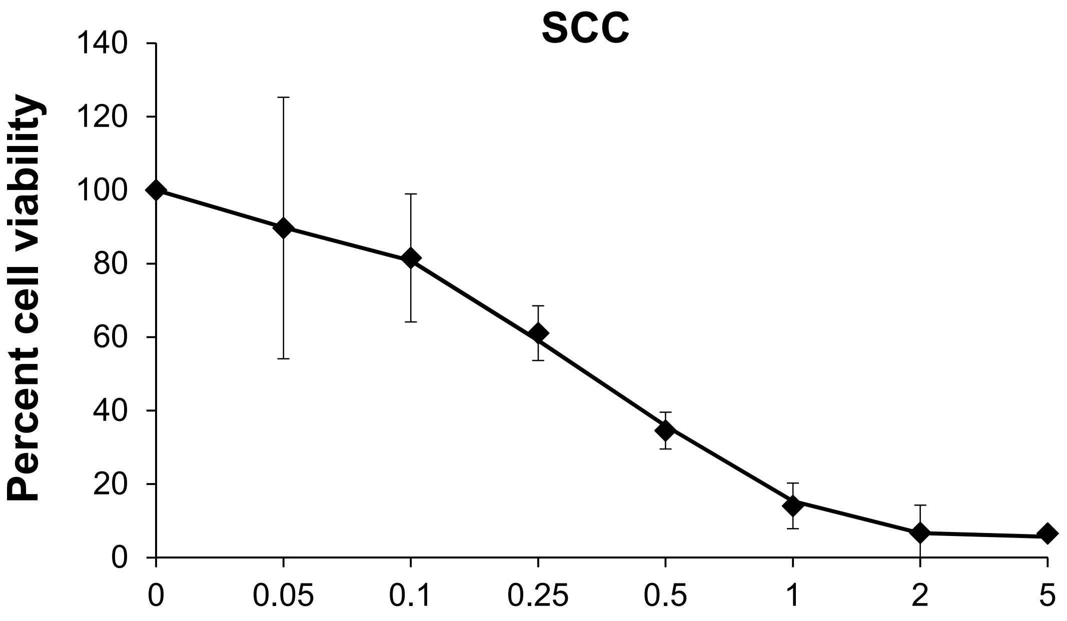

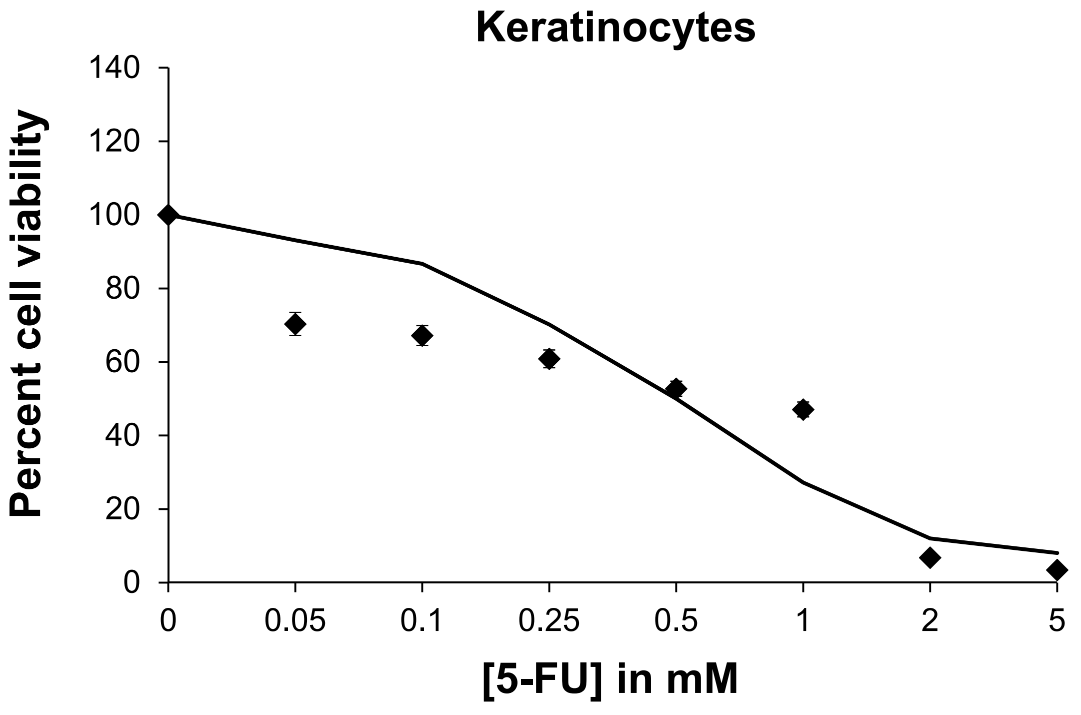

Figure S4. Dose-responses with treatment of 5-FU for 48-h continuous exposure.
